# Peripheral Plasma and Semen Cytokine Response to Zika Virus in Humans

**DOI:** 10.3201/eid2504.171886

**Published:** 2019-04

**Authors:** Jean-Michel Mansuy, Hicham El Costa, Jordi Gouilly, Catherine Mengelle, Christophe Pasquier, Guillaume Martin-Blondel, Jacques Izopet, Nabila Jabrane-Ferrat

**Affiliations:** Centre Hospitalier Universitaire de Toulouse, Toulouse, France (J.-M. Mansuy, H. El Costa, C. Mengelle, C. Pasquier, G. Martin-Blondel, J. Izopet);; INSERM U1043-CNRS UMR5282 Université Toulouse III, Toulouse (H. El Costa, J. Gouilly, G. Martin-Blondel, J. Izopet, N. Jabrane-Ferrat)

## Abstract

We assessed Zika virus RNA and select cytokine levels in semen, blood, and plasma samples from an infected patient in South America. Viral RNA was detected in semen >2 months after viremia clearance; cytokine profiles differed in semen and plasma. After viremia, Zika virus appears to become compartmentalized in the male reproductive tract.

Before the 2015–2016 outbreak, Zika virus infection had been associated with only mild symptoms. However, the outbreak revealed infection could cause severe clinical manifestations, particularly for fetuses and newborns (*1*). Furthermore, detection of replicative virus in semen and sexual transmission of the infection resulted in a paradigm shift in Zika virus virology (*2*,*3*). Several animal models were developed to study these phenomena, and studies revealed that Zika virus persistence within the male reproductive tract (MRT) results in diminished testosterone and oligospermia (*4*). However, because of complex ethical considerations, the consequences of infection on the MRT remain poorly understood (*5*).

To characterize infection in the MRT further, we conducted a longitudinal 6-month study examining Zika virus load and immunologic profile in blood, plasma, and semen in 1 man. The study patient was a 32-year-old immunocompetent white man with an asymptomatic Zika virus infection acquired in South America in January 2016; the control was a healthy 40-year-old white man without risk factors for acute or chronic infection who lived in the same area. We evaluated the concentrations of a select panel of cytokines, including innate immune mediators (interferon [IFN]–γ, interleukin [IL]–15, IFN-β); inflammatory factors (IL-6, IL-18, soluble intercellular adhesion molecule 1 [sICAM-1]); chemokines (CC-motif chemokine ligand [CCL] 3, CCL-4, CXC-motif chemokine ligand [CXCL] 1, CXCL-8, CXCL-10); hematopoietic factors (granulocyte colony–stimulating factor [G-CSF], granulocyte-macrophage colony–stimulating factor); the angiogenic factor vascular endothelial growth factor A (VEGF-A); and proteases (matrix metalloproteinase [MMP]–2, MMP-9). We quantified cytokines using ProcartaPlex Multiplex Assay (ebioscience, https://www.thermofisher.com).

At admission, the patient had moderate fever, maculopapular rash, myalgia, and arthralgia and recovered within a few days. He was HIV negative; dengue and chikungunya virus infections were ruled out using ELISA Diapro (Diagnostic Bioprobes Srl, https://www.diapro.it) and RealStar Dengue and Chikungunya RT-PCR Kit 2.0 (Altona Diagnostics, https://www.altona-diagnostics.com). The patient did not experience other genital or urinary tract infections during the study.

Two days after symptom onset, viral RNA was higher in semen (1.04 × 10^5^ copies/mL) than in blood (9.4 × 10^3^ copies/mL); RNA was detectable for up to 100 days in blood and 168 days in semen (Figure). Soluble factors secreted in response to infection could be stratified into 3 profiles. The first profile was a surge of proinflammatory cytokines in semen (IL-6, IFN-γ, CCL-3, CCL-4) and plasma (CCL-3, CXCL-10, VEGF-A) at early stages of infection. These cytokines returned to basal levels over time. The second profile (mainly observed in semen) involved decreased CXCL-1, CXCL-8, CXCL-10, and VEGF-A production during the first 50 days after infection, followed by a progressive return to reference ranges coinciding with viral clearance. The third profile involved sustained altered levels of factors in semen (IL-18, sICAM-1, G-CSF, MMP-2) and plasma (CCL-3, CXCL-1, sICAM-1, MMP-2, MMP-9), even after complete viral RNA clearance in plasma. Some factors were undetectable in plasma and semen samples (IL-15, IFN-β, granulocyte-macrophage colony–stimulating factor), and others were absent or barely detectable in plasma (IL-6, IFN-γ, CCL-4, CXCL-8, G-CSF).

**Figure:**
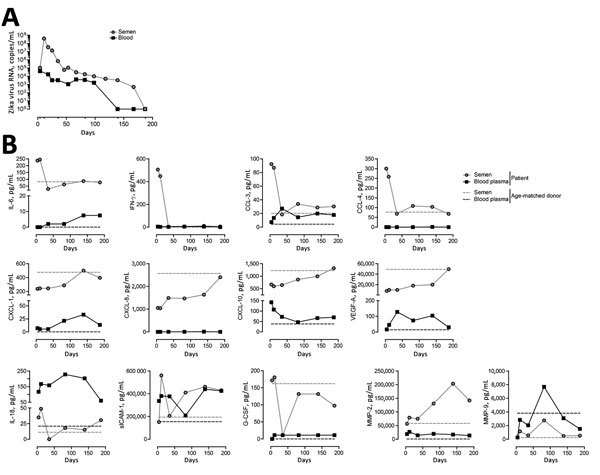
Kinetics of Zika virus replication and cytokine secretion in semen, blood, and plasma samples from a Zika virus–infected patient, France, 2016. A) Kinetics of Zika virus RNA detection in whole blood and semen quantified by real-time reverse transcription PCR. B) Quantification of soluble factors in the blood plasma and semen at 4, 11, 35, 82, 139, and 187 days after symptom onset. Dashed lines represent baseline levels of soluble factors quantified in blood plasma and semen obtained from an age-matched healthy donor. CCL, CC-motif chemokine ligand; CXCL, CXC-motif chemokine ligand; G-CSF, granulocyte colony–stimulating factor; IFN-γ, interferon γ; IL, interleukin; MMP, matrix metalloproteinase; sICAM-1, soluble intercellular adhesion molecule 1; VEGF-A, vascular endothelial growth factor A.

Our data revealed higher amounts of and more prolonged viral RNA shedding in semen than in blood. Zika virus infection altered cytokine secretion and induced distinct profiles in the plasma and semen; changes in the semen were more prominent. The discrepancies in kinetics and concentrations of cytokines and virus titers between semen and plasma suggest virus compartmentalization.

Cytokines produced in the MRT reflect local immune status, organ function, and tissue damage. The high levels of proinflammatory factors in semen samples from this patient illustrate an exacerbated local immune response established to control Zika virus infection. This response might lead to recruitment of more host and inflammatory cells that further amplify viral replication and organ injury (*6*). Downregulation of several factors highlights the damage. For instance, the VEGF-A levels mirror the impairment of spermatogonia, primary spermatocytes, and Sertoli cells upon Zika virus infection (*4*). However, the decrease in CXCL-1, CXCL-8, and CXCL-10 levels in semen during infection could indicate a local immunosuppressive state induced by infection, limiting immune cell infiltration in the MRT and potentially virus dissemination throughout the body.

The different kinetics of virus replication and cytokine secretion in semen samples raises questions about the semen secretome in cases of couples aiming for conception and the necessity to extend the convalescence period beyond Zika disease recovery. In fact, at high concentration, most of these factors might alter the integrity of the mucosal barriers within the female reproductive tract and increase a woman’s susceptibility to infection (*7*). They might also promote peroxidation and affect sperm function, potentially resulting in infertility (*8*).

The limitations of our study include small sample size. Further investigations with a larger cohort of patients and controls warranted.

In summary, a profound disruption in the cytokine network is evident in plasma and semen starting at the earliest stage of Zika virus infection and is maintained over time even after viral clearance. Studies to characterize the mechanism involved in the establishment of compartmentalization and develop efficient antiviral therapies that interfere with virus replication in the MRT are needed.
